# Aggressive approach for spontaneous pneumothorax treatment in children with Marfan syndrome?

**DOI:** 10.3389/fped.2023.1301902

**Published:** 2023-12-18

**Authors:** Angelo Zarfati, Simone Frediani, Valerio Pardi, Ivan Pietro Aloi, Silvia Madafferi, Antonella Accinni, Arianna Bertocchini, Alessandro Inserra

**Affiliations:** General and Thoracic Pediatric Surgery Unit, Bambino Gesù Children’s Hospital, IRCCS, Rome, Italy

**Keywords:** Marfan syndrome, children, pneumothorax, thoracic drainage, thorax

## Abstract

**Background and objectives:**

Marfan syndrome (MS) is a systemic disease of connective tissues consisting of a variable combination of anomalies. These patients have an increased risk of spontaneous pneumothorax (SP). However, there is a scarcity of pediatric literature on management, and no specific guidelines exist. Our aim was to analyze the management of spontaneous pneumothorax in children and adolescents with Marfan syndrome, comparing syndromic and non-syndromic patients.

**Methods:**

Retrospective analysis of pediatric patients (18 years) with SP diagnosed at our tertiary pediatric hospital (January 10–June 22), with special emphasis on diagnosis, treatment, and follow-up (FU).

**Results:**

Sixty-six patients with SP were identified, with nine (13%) having MS. In terms of baseline, there were no significant differences between the groups (age, sex, asthma, symptoms, and side, first-line treatment and hospitalization length). Overall, Marfan patients had significantly more first-line treatment failures requiring additional surgery, as well as more contralateral occurrences and the need for surgery/chest drain during the follow-up. Instead, conservative management resulted in significantly more ipsilateral recurrences and the need for surgery/chest drain in Marfan patients than controls during the follow-up.

**Conclusions:**

Treatment failure, contralateral occurrence, ipsilateral recurrence, and the need for surgery/chest drain during follow-up make management of patients with Marfan syndrome and spontaneous pneumothorax more difficult. In patients with a diagnosed MS a more aggressive first-line management should be considered, bearing in mind the higher risks of this population.

## Introduction

Marfan syndrome (MS) is a genetic (autosomal dominant) systemic connective tissue disease with no gender or ethnic preference (1). A prevalence of 1/10,000 and 1/15,000 has been reported (1–3). The disorder is caused by a FIBRILLIN-1 mutation and is characterized by a variable combination of different multisystem anomalies (2–4). Even though the syndrome has a wide range of clinical manifestations, it is most commonly associated with cardiac, vascular, ocular, and musculoskeletal symptoms (1). However, up to one in every ten patients may have some type of lung or respiratory anomaly (1, 2). Several respiratory disorders associated with Marfan syndrome have been identified, such as chronic bronchitis, lung emphysema, pneumothorax, bronchiectasis, thoracic musculoskeletal malformations, and sleep problems (1, 4, 5). Despite its rarity, spontaneous pneumothorax has been included in the Ghent criteria for diagnosing the syndrome (6). Marfan patients have an increased risk of spontaneous pneumothorax and present earlier than their peers (7). The Marfan's increased risk of pneumothorax has been likened to apical blebs, bullae, anomalous connective tissue of the lung parenchyma, or excessive stress in the lung apices induced by tall habitus (5). The recommended management of a first episode of spontaneous pneumothorax in adult Marfan patients is the same as in the general population (1). However, evidence for this unusual combination is limited, particularly in children. There are no age-specific practice guidelines or recommendations available currently. We've always suspected that managing SP in Marfan patients is more difficult than in the general population, both during the initial episode and during follow-up. Furthermore, a recent Japanese study confirmed our suspicions, reporting a lower response and worse outcomes for Marfan patients treated conservatively (7). Indeed, in this extremely select group of pediatric patients, the Authors recommended surgery as first-line treatment for spontaneous pneumothorax.

The aim of the study was to analyze our experience with the management of the first episode of spontaneous pneumothorax in children and adolescents with Marfan syndrome, comparing syndromic and non-syndromic patients.

## Methods

A retrospective analysis of pediatric patients (<18 years-old) treated for a first episode of primary spontaneous pneumothorax at our tertiary center (General and Thoracic Pediatric Surgery Unit, Bambino Gesù Children's Hospital, IRCCS, Rome, Italy) between 2010 and 2022 was undertaken. Clinical, imaging, and surgical data, as well as follow-up and outcomes, were extracted from medical charts and radiological systems. Pairwise comparisons among Marfan and non-syndromic (control) patients were realized. Exclusion criteria include missing or incomplete charts/data, no follow-up, and being over the age of 18 at the time of the first episode. The sample size was determined by the number of cases managed. Patients referred locally and nationally were included in the study.

The details and methodology of management of spontaneous pneumothorax in our institution have been recently published (8). Patients were defined as asymptomatic in the absence of symptoms, paucisymptomatic in case of mild chest pain, cough, or mild dyspnea, and highly symptomatic in the case of severe pain or dyspnea. The failure of the first-line management was defined as the need for surgery for a spontaneous pneumothorax refractory or persistent to the treatment. At our institution we use digital thoracic drainage systems in the management of spontaneous pneumothorax (9). Our postoperative chest tube management and discharge criteria were as follows: if there is no evidence of an air leak, the chest tube is closed. The next day, a chest x-ray is obtained to rule out any persistent pneumothorax (12–24 h from closure). If there is no or very little persistent pneumothorax, the tube is removed. In the absence of complications, patients are discharged after the chest tube is removed. During the follow-up, the ipsilateral recurrences and contralateral occurrences were analyzed separately.

Categorical variables are reported as absolute and relative frequencies, and continuous variables as median and range. Groups were compared using the *χ*^2^ test or the Fisher exact test for categorical variables, as appropriate. For continuous variables, differences between groups were established with a non-parametric test, *U* Mann–Whitney test. All *p*-values were two-sided, and a value <0.05 was considered significant.

This study did not receive any specific grants from funding agencies in the public, commercial, or nonprofit sectors. The authors have no financial relationships or conflicts of interest to disclose.

The present study received authorization for publication from the scientific board in the authors' institution.

## Results

During a 12-year study period, our tertiary center identified 66 pediatric patients with their first episode of spontaneous pneumothorax (5.5/year). Marfan Syndrome (0.75/year) affected nine patients (13%) of the analyzed population. Table 1 details the groups' baselines and diagnoses. Marfan and control showed no statistically significant differences in all baseline and diagnosis characteristics, such as age at first episode [median 15.4 years (range 11.5–17.0 years) vs. 15.4 years (range 2.3–17.9 years), *p* = 0.303], male sex (88% vs. 75%, *p* = 0.671), history of asthma (0% vs. 12%, *p* = 0.580), highly symptomatic patients (11% vs. 9%, *p* = 1.000), and side (Left 44% vs. 63%, *p* = 0.300; Right 55% vs. 35%, *p* = 0.282; Bilateral 0% vs. 1%, *p* = 1.000).

**Table 1:** Baseline and diagnosis of the study populations.

		Total (*n* = 66)	Marfan (*n* = 9)	Control (*n* = 57)	*p*-value
Median age (1st ep.) (range)		15.4 (2.3–17.9)	15.4 (11.5–17.0)	15.4 (2.3–17.9)	0.303
Males		77% (51)	88% (8)	75% (43)	0.671
Asthma		10% (7)	0% (0)	12% (7)	0.580
Highly symptomatic (1st ep.)		10% (7)	11% (1)	9% (6)	1.000
Side	*Left*	60% (40)	44% (4)	63% (36)	0.300
	*Right*	37% (25)	55% (5)	35% (20)	0.282
	*Bilateral*	1% (1)	0% (0)	1% (1)	1.000

Table 2 details the characteristics and outcomes of management at the first episode of spontaneous pneumothorax and follow-up in the various groups. There were no statistically significant differences in the type of first line management between Marfan and control patients (Conservative 44% vs. 56%, *p* = 0.720; Operative 56% vs. 44%, *p* = 0.720). Among the cases that underwent a first-line operative management, the groups were similar even regarding the type of operative treatment (Drainage 44% vs. 36%, *p* = 0.720; Surgery 11% vs. 7%, *p* = 0.531). The patients with Marfan syndrome showed significantly more first-line treatment failures needing further surgery [44% (4) vs. 14% (8), *p* = 0.049]. However, the median hospitalization at first episode was similar among the groups [5 days (range 2–15) vs. 6 days (range 0–45), *p* = 0.603].

**Table 2:** First-line management, outcomes, and follow-up.

			Total (*n* = 66)	Marfan (*n* = 9)	Control (*n* = 57)	*p*-value
First-line management	Conservative		54% (36)	44% (4)	56% (32)	0.720
	Operative	Overall operative	45% (30)	56% (5)	44% (25)	0.720
		*Drainage*	37% (25)	44% (4)	36% (21)	0.720
		*Surgery*	7% (5)	11% (1)	7% (4)	0.531
Median days of hospitalization (range)			6 (0–45)	5 (2–15)	6 (0–45)	0.603
1st Treatment failures needing further surgery			**18%** **(12)**	**44%** **(4)**	**14%** **(8)**	**0.049**
Ipsilateral recurrence			36% (24)	55% (5)	33% (19)	0.267
Contralateral occurrence			**15%** **(10)**	**55%** **(5)**	**8%** **(5)**	**0.002**
≥1 surgery or chest drain during the FU			**34%** **(23)**	**66%** **(6)**	**29%** **(17)**	**0.050**

The patients were followed-up for a median of 1.1 years since the first episode of spontaneous pneumothorax (range 0.1–12 years) and until a median age of 17.2 years (range 5.5–24.5 years). During the follow-up Marfan and control patients showed no significant differences regarding the incidence of ipsilateral recurrence (55% vs. 33%, *p* = 0.267). However, the MS group experienced significantly more contralateral occurrence (55% vs. 8%, *p* = 0.002). Furthermore, the cases requiring at least one surgery, or a chest drain during the follow-up were significantly more in the Marfan group (66% vs. 29%, *p* = 0.050).

Table 3 shows a comparison of the outcomes of conservative first-line management in the groups. The groups had no significant differences regarding first line treatment failure [25% (1) vs. 6% (2), *p* = 0.305]. However, patients with an ipsilateral recurrence [75% (3) vs. 21% (7), *p* = 0.050] and the cases requiring at least one surgery, or a chest drain during the follow-up were significantly more in the Marfan group [75% (3) vs. 12% (4), *p* = 0.017].

**Table 3:** Comparison of the outcomes of the conservative first-line management in the groups.

Conservative	Total (*n* = 36)	Marfan (*n* = 4)	Control (*n* = 32)	*p*-value
1st line Treatment failure	8% (3)	25% (1)	6% (2)	0.305
Ipsilateral recurrence	**27%** **(10)**	**75%** **(3)**	**21%** **(7)**	**0** **.** **050**
≥1 surgery or chest drain during the FU	**19%** **(7)**	**75%** **(3)**	**12%** **(4)**	**0** **.** **017**

Table 4 shows a comparison of the outcomes of operative first-line management in the groups. The subgroups were similar regarding first-line treatment failure [60% (3) vs. 24% (6), *p* = 0.143], ipsilateral recurrence [40% (2) vs. 48% (12), *p* = 1.000], and cases requiring at least one surgery, or a chest drain during the follow-up [60% (3) vs. 52% (13), *p* = 1.000].

**Table 4:** Comparison of the outcomes of the operative first-line management in the groups.

Operative	Total (*n* = 30)	Marfan (*n* = 5)	Control (*n* = 25)	*p*-value
1st line treatment failure	30% (9)	60% (3)	24% (6)	0.143
Ipsilateral recurrence	46% (14)	40% (2)	48% (12)	1.000
≥1 surgery or chest drain during FU	53% (16)	60% (3)	52% (13)	1.000

## Discussion

Marfan patients constitute a non negligeable portion of spontaneous pneumothoraxes treated at pediatric centers. Marfan syndrome was found in 13% of patients with a first episode of SP in our study. However, there is very little evidence for this unusual combination, particularly in children and adolescents. There are no age-specific practice guidelines or recommendations available currently. Our experience does not support the reported earlier presentation of spontaneous pneumothorax in syndromic patients compared to their peers (7). Indeed, Marfan and controls were about the same age when they had their first episode of spontaneous pneumothorax. The population presented in the study confirmed the low treatment response and high rate of incidence of recurrence reported in the existing literature (7). Indeed, we group experienced overall 44% of treatment failure, 55% of ipsilateral recurrences, 55% of contralateral occurrences, and 66% of patients needed at least one surgery or chest drain during the follow-up. For all these outcomes except ipsilateral recurrences, Marfan patients had a significantly an overall higher incidence than control group. Furthermore, among the cases treated conservatively, MS experienced 75% of ipsilateral recurrence and 75% of need for surgery during the follow-up. This was significantly higher than non-syndromic group. Instead, among the cases treated operatively, the incidence of ipsilateral recurrence and need for surgery during the follow-up was similar between syndromic and control groups.

We found only a similar series existing in pediatric patients in the contemporary English literature, that is the above mentioned study from Japan (7). Sunochi et al. focused specifically on the challenges of managing spontaneous pneumothorax in pediatric patients with Marfan syndrome. The Japanese group, like the current study, reported a low response rate in conservatively managed Marfan patients with spontaneous pneumothorax. The treatment failure was 57% when conservative management, and 13% for operative. Furthermore, 67% of patients with successful conservative management experienced an ipsilateral relapse. This data is almost entirely consistent with our findings in this regard. Therefore, this group supported the role of an early first-line surgical treatment in this population. However, compared to the present experience, the reported failure was apparently relatively higher in the conservative group (57% vs. 25%) and apparently lower in the operative group (13% vs. 60%). This disparity with the Japanese group can be explained by differences in methodology. Indeed, in our institution we considered the placement of a chest tube among the operative treatment. Instead, the authors of above-mentioned series included it as part of the conservative management. As a result, comparisons between the series in this regard are risky and potentially misleading. Even if the studies are difficult or impossible to compare, we believed that the above-mentioned study from Sunochi et al. was worth mentioning in the discussion of our paper. Indeed, in the authors' best knowledge, this study represents the only published experience on the specific management of pneumothorax in Marfan pediatric patients.

Our institution has recently developed a management protocol for spontaneous pneumothorax in children and adolescents (8). The protocol is based on the authors' expertise and experience, as well as recent literature (10–13). At the first episode of SP in the pediatric population (excluding neonatal pneumothorax), management should be guided by the clinical status and severity of symptoms. SP patients who are asymptomatic or pauci-symptomatic should be admitted to the hospital and treated conservatively with oxygen and pain relievers. It the evolution is favorable, with decrease of the pneumothorax, the patient can be discharged. In 7–10 days, the patient is seen for a clinical and radiological outpatient check. Patients should have a thoracoscopic exploration if they have severe symptoms during the first episode, or if the pneumothorax worsens or persists. In the event of a recurrence, patients should be admitted to the hospital and treated with oxygen therapy and thoracoscopy. Furthermore, during their hospital stay, these patients should have a multidisciplinary examination for comorbidities, which at our institution includes a genetic, cardiologic, and ophthalmologic work-up to rule out collagenopathies such as Marfan syndrome. Patients who present with severe symptoms should be hospitalized and treated with oxygen and thoracoscopic exploration. In any case where a tensile pneumothorax is suspected, a prompt chest drain should be placed. The pleural space should be thoroughly explored each time a VATS is performed. A bullectomy should be performed in cases of blebs. In the absence of obvious blebs, a talcage should be considered. A post-operative chest tube should be introduced in any case. Before repeating a VATS exploration in cases of post-operative pneumothorax persistence or ipsilateral recurrence, a chest CT should be considered to assess any anatomical (residual blebs, etc.) or pathological (associated anomalies, etc.) etiology. However, in the light of our findings of the present study and the existing evidence this specific setting seems to not be suitable for Marfan patients (7). Indeed, more aggressive first-line management should be considered when a spontaneous pneumothorax occurs in a patient with a diagnosed Marfan syndrome. Indeed, even in minor or asymptomatic pneumothorax, thoracoscopic exploration with eventual bullectomy and/or talcage should be considered from the first episode.

The current study has some limitations that must be acknowledged. The present experience's strengths must be qualified by its retrospective and monocentric design. It should be noted that we're reporting a relatively large population of this exceedingly rare combination of spontaneous pneumothorax in MS in pediatric patients. The syndrome is extremely rare, with an estimated prevalence of 1/10,000–1/15,000. Moreover, even though Marfan has a wide range of clinical manifestations, it is most associated with cardiac, vascular, ocular, and musculoskeletal symptoms. Indeed, only one in every ten patients may have some type of lung or respiratory anomaly. Moreover, no randomization was realized. Further research, possibly prospective and/or multicentric, is required to validate our findings.

## Conclusions

Management of patients with Marfan syndrome and spontaneous pneumothorax is complicated by treatment failure, contralateral occurrence, ipsilateral recurrences, and the need for surgery/chest drain during follow-up. During the follow-up, conservatively managed Marfan patients have a higher incidence of ipsilateral recurrence and the need for surgery. Given the higher risks of this population, a more aggressive first-line management should be considered, even for little or asymptomatic SP.

## Data Availability

The original contributions presented in the study are included in the article/Supplementary Material, further inquiries can be directed to the corresponding author.
